# Depression, Comorbidities, and Prescriptions of Antidepressants in a German Network of GPs and Specialists with Subspecialisation in Anthroposophic Medicine: A Longitudinal Observational Study

**DOI:** 10.1155/2012/508623

**Published:** 2012-12-06

**Authors:** Elke Jeschke, Thomas Ostermann, Horst C. Vollmar, Manuela Tabali, Harald Matthes

**Affiliations:** ^1^Havelhoehe Research Institute, Kladower Damm 221, 14089 Berlin, Germany; ^2^Center for Integrative Medicine, University of Witten/Herdecke, Gerhard-Kienle-Weg 4, 58313 Herdecke, Germany; ^3^Department of General Practice, University of Düsseldorf, Moorenstraße 5, 40225 Düsseldorf, Germany; ^4^Institute for General Practice and Family Medicine, University of Witten/Herdecke, Alfred-Herrhausen-Straße 50, 58448 Witten, Germany

## Abstract

*Background*. Depression is a major reason for counselling in primary care. Our study aims at evaluating pharmacological treatment strategies among physicians specialised in anthroposophic medicine (AM). *Methods*. From 2004 to 2008, twenty-two German primary care AM-physicians participated in this prospective, multicentre observational study. Multiple logistic regression was used to determine factors associated with a prescription of any antidepressant medication. *Results*. A total of 2444 patients with depression were included (mean age: 49.1 years (SD: 15.4); 77.3% female). 2645 prescriptions of antidepressants for 833 patients were reported. Phytotherapeutic preparations from *Hypericum perforatum* were the most frequently prescribed antidepressants over all (44.6% of all antidepressants), followed by amitriptyline (16.1%). The likelihood of receiving an antidepressant medication did not depend on comorbidity after controlling for age, gender, physician specialisation, and type of depression (adjusted OR (AOR) = 1.01; CI: 0.81–1.26). Patients who had cancer were significantly less likely to be prescribed an antidepressant medication than those who had no cancer (AOR = 0.75; CI: 0.57–0.97). *Conclusion*. This study provides a comprehensive analysis of everyday practice for the treatment of depression in AM -physicians. Further analysis regarding the occurrence of critical combinations is of high interest to health services research.

## 1. Background

Depression is one of the three leading causes of disease burden worldwide strongly correlated with increased morbidity and a major reason for counselling and primary patient care [1, 2]. Depending on the study origin and setting, the prevalence of depression in the general population is estimated between 10% and 25% in females and 5–12% in males with a one-year incidence rate of approximately 2% [3–5]. Thus, early detection and treatment of depression is a major task for health care policy makers. Due to increasing patient numbers and the development of new antidepressive drugs, family physicians today play an important role in the treatment of depressed patients [6, 7]. Although approximately 40% of patients with depression still remain untreated, those patients who decide to consult a therapist are more likely to see a family physician than a psychiatric specialist for both diagnosis and treatment. This is quite important as knowledge and accuracy of nonpsychiatric physicians in treating depression have a great influence the outcome of the illness. 

Although research has significantly advanced in the last years, and depression is now generally more acknowledged as an important factor in primary care, patients, relatives, and physicians still have reservations and prejudices against pharmaco- or psychotherapy, which may aggravate a sufficient and individualized treatment of depression in primary care and may account for insufficient treatment of depressive symptoms [7]. Studies have shown that only a small amount of primary care patients diagnosed with depression receive appropriate care, which may further lead to poor treatment outcome and increased mortality [4, 7]. 

Accordingly, national guidelines about depression treatment in primary care are a key area of public policy. In Germany that is, the development of a German National Disease Management Guideline (DM-CPG) for depression was induced to increase transparency and improve patient care [6, 8, 9]. 

With respect to complementary and alternative medicine (CAM), guidelines from Germany, Canada, and Switzerland have listed the phytopharmaceutical preparation of *Hypericum perforatum* [6, 10, 11] which is traditionally used quite frequently for mild and moderate depressions. Moreover its effectiveness for unipolar depressive episodes was shown in systematic reviews and meta-analyses [12, 13]. But also other drugs from phytotherapy or homoeopathy may have a potential in the treatment of depression [14, 15]. However, the prescribing of antidepressants is influenced by physician—as well as patient–related factors, and less is known about prescribing habits of physicians in primary care particularly of those being specialized in CAM. 

The present study, thus, aims to analyse prescribing patterns in a network of GPs and specialists with subspecialisation in anthroposophic medicine (AM) for patients who experienced a new episode of depression and to investigate conformity and variations in antidepressant prescriptions. It was hypothesized that (a) *Hypericum perforatum* was the most frequently prescribed antidepressant and (b) that patients with co-morbidities were more likely prescribed any antidepressant medication. 

## 2. Methods

Physicians for the EvaMed Network were recruited through the German National Association of Anthroposophic Physicians (GAÄD) in 2004 [16]. At that time, 118,085 primary care physicians were practising in Germany. Of those, 626 (0.5%) primary care physicians were members of the GAÄD. For a physician to be eligible to participate in the study, his or her medical practice had to meet a number of technical requirements, including the presence of a special computerized patient documentation system (DocExpert, DocConcept, TurboMed, Duria, PDE-Top, and Medistar), a local area network (LAN) connection, and Microsoft Windows and Internet Explorer (i.e., as client software). From the 626 physicians of the GAÄD, 362 (57.8%) met these criteria based on self-reported information and were contacted. Physicians were required to give their informed consent to participate in the EvaMed Network and to report all detected serious ADRs (definition provided below “data collection and classification of ADRs”) to the EvaMed Network. A total of 38 physicians from 12 of the 16 federal German states finally agreed to participate in EvaMed, covering 6.1% of the overall primary care physicians of the GAÄD [17]. They all had practised for at least five years in primary care in addition to completing training in anthroposophic medicine.

For our study, 16 physicians specialized in paediatrics, dermatology, and gynaecology were excluded from the study which led to 22 physicians who participated in this study. 

The present study is based on secondary data provided by the physicians. As such, the recommendations for good practice in secondary data analysis (e.g., anonymization of data on prescriptions and diagnoses) were developed by the German Working Group on the Collection and Use of Secondary Data were applied in full [18]. 

Patients were included if they had at least one diagnosis of depression according to the 10th revision of the International Classification of Diseases (ICD10: F32 or F33) during a 5-year study period (01·01·2004–01·01·2009). Patients were excluded if patients were <18 years of age. Patients were also excluded if there was no new diagnosis of depression during the study period. “New diagnosis” of depression was operationally defined as having no diagnosis of depression before and no prescription of any antidepressant medication during the 6 months preceding the index diagnosis. Patients who had no office visit before the index depression diagnosis were also excluded because it was not able to distinguish, whether the index diagnosis represented either a new diagnosis of depression or the entry of an established diagnosis of depression for a new patient. Finally, we also excluded patients with a recorded diagnosis of mania (F30), bipolar disorder (F31), or schizophrenia (F20) because it was thought that these patients would be treated differently. 

During the study, physicians continued to follow their routine documentation procedures, recording diagnoses, and all prescriptions for each consecutive patient using their existing computerized patient documentation system. These data were exported to the QuaDoSta postgreSQL database hosted in each practice [19]. Physicians used a browser-based interface to match individual diagnoses with the corresponding drugs or remedies that had been prescribed. Prescribed drugs were documented using the German National Drug Code. Diagnoses were coded according to the 10th revision of the International Classification of Diseases (ICD-10). 

Depression was classified as “depressive episode” (ICD10: F32) or “recurrent depressive disorder” (ICD10: F33). Co-morbidities were classified as coronary heart disease (ICD10: I20-I25), cerebrovascular disease (ICD10: I60-I69), diabetes mellitus (ICD10: E10-E14), cancer (ICD10: C00-C97), congestive heart failure (ICD10: I50), and chronic obstructive pulmonary disease (COPD; ICD10: J44). Multi-morbidity was considered if a patient had at least two co-morbidities.

Study investigators identified all drugs and remedies prescribed for depression. Each substance was classified using the Anatomical Therapeutic Chemical Index German version (ATC). Antidepressant medication was clustered into non-selective monoamine reuptake inhibitors (NSMRIs; ATC: N06AA), selective serotonin reuptake inhibitors (SSRIs; ATC: N06AB), monoamine oxidase inhibitors (MAOIs; ATC: N06AF), non-selective monoamine oxidase A inhibitors (ATCs: N06AG), other antidepressants (e.g., bupropion, mirtazapine, and nefazodone; ATC: N06AX), and phytotherapeutic antidepressants (N06APs). 

Statistical analysis was performed with SPSS 18.0 for Windows. Descriptive analysis was used to determine prescription rates. Means and standard deviations (SDs) were calculated for continuous data. In cases where data were not normally distributed, medians and interquartile ranges (IQRs) were reported. Subgroup analyses of prescribing rates were performed for patient age (18–39 years, 40–59 years, and 60 years and older), gender, and co-morbidities. The two-tailed chi-square test was used to analyse differences in prescription rates. A *P* value of less than 0.05 was regarded as indicating a statistically significant difference. 

Odds ratios (ORs) and 95% confidence intervals (CIs) were calculated using multiple logistic regression with any antidepressant being prescribed medication as the outcome variable. For each outcome ORs were calculated for patients who had and did not have each of the co-morbidities as well as for who had and did not have any of the 6 co-morbidities. After calculating unadjusted OR, two models including potential confounders were determined. Model 1 was controlled for patient age and gender, and model 2 was controlled for patient age, gender as well as for physicians' gender and specialisation and type of depression. Patient age was introduced in the model as a continuous variable. 

## 3. Results

Of the 22 physicians, 17 were GPs (77%) and 5 were specialists working as GPs (23%). The participating physicians did not differ significantly from the overall population of physicians certified in anthroposophy in Germany (*n* = 362) in terms of age (mean = 49.4; SD = 6.3 years versus mean = 47.5; SD = 6.1 years; *P* = 0.709) or gender (60.0% versus 62.2% men; *P* = 0.917) and were only slightly younger and consisted of a similar percentage of women compared to all office-based physicians in Germany (mean 52.0 years; 61.2% men) [20].

**Figure 1:**
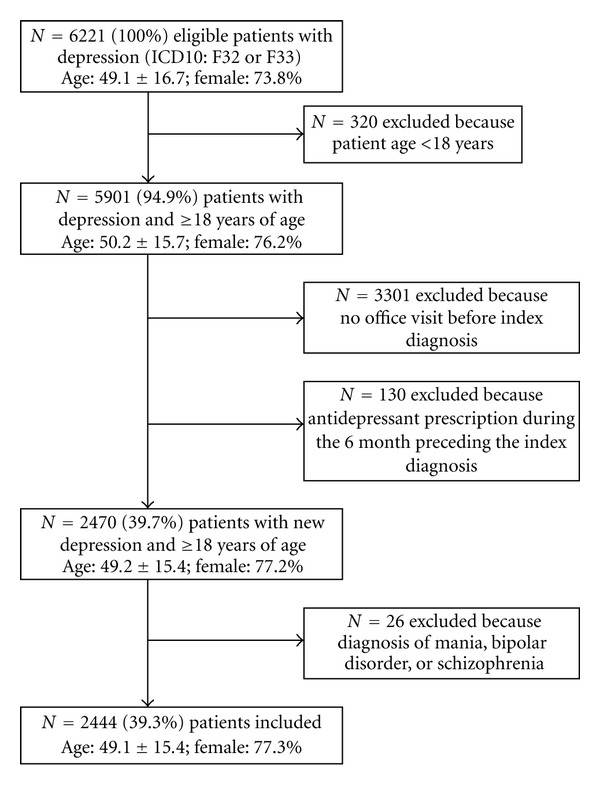
Flow chart of the inclusion process.

During the 5-year study period, a total of 2444 patients with depression were included. The inclusion process is shown in Figure 1. 73.4% of all patients were treated by a GP (*n* = 1793), 17.9% by an internist (*n* = 437), and the remaining 8.8% of the patients were treated by a neurologist (*n* = 214). 77.3% of the patients were female (*n* = 1889). The mean age of the patients was 49.1years (SD = 15.4). Altogether, 26.8% of the patients were 18–39 years (*n* = 656), 49.8% were 40–59 years (*n* = 1218), and 23.3% were 60 years or older (*n* = 570). Depression was classified as depressive episode (88.3%) and recurrent depressive disorder (11.7%). There was no significant difference according to type of depression and age group (*P* = 0.789) or gender (*P* = 0.658). 

In total, 8.3% of all patients (*n* = 204) had two or more co-morbidities and were, therefore, classified as multi-morbid. The most frequent co-morbidities were cancer (14.4% of all patients), coronary heart disease (8.3%), and diabetes mellitus (7.1%). Table 1 provides a detailed overview of the co-morbidities of the participating patients according to patient age and gender. 

Overall, 833 patients were prescribed an antidepressant medication, representing 33.9% of patients who experienced a new episode of depression. In total, 2645 prescriptions of antidepressants for these patients were reported. They were nearly uniformly distributed over the four quarters (1st quarter: 630 (23.8%), 2nd quarter: 645 (24.4%), 3rd quarter: 616 (23.3%), and 4th quarter: 754 (28.5%)).

Table 2 gives an overview of the prescribed antidepressants. Phytotherapeutic preparations of *Hypericum perforatum* were the most frequently prescribed antidepressants over all (44.6% of all antidepressants). The most common class of conventional antidepressants prescribed was the NSMRI class, and amitriptyline was the most commonly prescribed individual medication. 

Table 3 gives a detail overview of the included patients according to antidepressant medication. Phytotherapeutic preparations from *Hypericum perforatum* were prescribed to 539 of 833 patients with any antidepressant medication (64.7%), followed by NSMRI (28.2%), and SSRI (16.8%). The proportion of patients with antidepressant medication was especially high among neurologists (76.2%). The proportion of patients being prescribed any antidepressant medication increased with patient age from 27.7% of patients under 40 years to 44.4% of patients of 60 years or older. Patients with multi-morbidity were more likely to receive an antidepressant than patients without co-morbidity (47.1% versus 32.9%; *P* = 0.016 chi-square test). The differences in age and co-morbidities were only due to conventional antidepressants, especially to NSMRI, whereas there was no difference in the prescription rates of phytotherapeutic preparations from *Hypericum perforatum*. 

As shown in Table 4, the likelihood of being prescribed an antidepressant medication was not significantly different for persons who had a co-morbid condition compared with those who did not have a co-morbid medical condition after controlling for age and gender (model 1: adjusted OR = 0.88; CI: 0.71–1.09) and after controlling for further potential confounder (model 2: adjusted OR = 1.01; CI: 0.81–1.26). But there were significant differences according to the presence or absence of the individual co-morbidities. The adjusted OR for receiving any antidepressant medication was greater than 1 for the co-morbidity cerebrovascular disease (model 1: adjusted OR = 1.78; CI: 1.16–2.74; model 2: adjusted OR = 1.76; CI: 1.12–2.76). Patients who had cancer were significantly less likely to be prescribed an antidepressant medication than those who had no cancer (model 1: adjusted OR = 0.65; CI: 0.51–0.84; model 2: adjusted OR = 0.75; CI: 0.57–0.97). Finally model 2 also indicated OR < 1 for the co-morbidities heart failure and COPD.

Our data, however, suggest an increase in antidepressant medication over the time of the study period. While in 2004, a total of 579 patient were prescribed 360 antidepressant drugs (mean 0.62), the amount of prescribed antidepressants almost doubled to a mean of 1.28 in 2008 (376 patients with 483 prescriptions). 

## 4. Discussion

In this paper, we presented the results of a secondary data analysis of electronic health record data from the EvaMed-Network, a German network of physicians with a subspecialisation in anthroposophic medicine [16, 17, 19] which aims at improving clinical practice by collecting prescription and ADR data. 

In the current study, 2444 patients with a first diagnosis of depression fitted the inclusion criteria. A proportion of 8.3% of them were multi-morbid with more than two diagnoses. 33.9% of the patients received an antidepressant medication. The proportion of patients with medications is much less compared to the findings of, for example, 51.9% by Robinson et al., 76.1%, and accordingly 77.4% by Gill and colleagues in 2008 and 2010 respectively 2010 [21–23]. This is even more of relevance as our patients received more complementary drug medication with phytotherapeutic preparations from *Hypericum perforatum* being the most prescribed drug over all. Within our study period, the number of psychiatric diseases and in particular depressive disorders in Germany significantly rose which is reflected in the data of prescription costs of antidepressants which according to health insurance data rose from 5 Mio. Euro in 2000 up to 14.5 Mio. Euro in 2009 [24]. Published data also suggest a higher proportion of female patients receiving such medication [25, 26]. Both of these are strongly supported by our findings with three of four medicated patients being female and a doubling in the prescribed drugs per patient from 2004 to 2008. 

To improve the situation of people with depression in Germany, a first measure was the implementation of the German Disease Management Guideline (DMG-CPG) for depression [6, 8, 9]. The increased prescriptions of new antidepressive pharmacotherapies like SSRIs nowadays is critically discussed within the scientific community [26, 27]. One of their major concerns is the unjustified medication of mild and potentially self limiting depressive episodes with expensive medications with a high potential of adverse drug reactions.

Our study also gives data on the prescription of NSMRI (28.2%) and SSRI (16.8%) which is considerably below the German standard. One reason might be the compensation of such drug classes by the use of complementary drug therapies like *Hypericum perforatum.*

Several publication on prescriptions [24] state that citalopram, mirtazapin and amitriptylin are the most common and popular remedies for depression. We also found these three remedies to be the most often prescribed conventional drugs. However, we can not tell why the ranking in our study is the other way round (Amitriptylin, Mirtazapine followed by Citalopram). This may be due to the comparably longer time frame of our study or to the different sample of physicians. One explanation might also be that Amitriptylin is the “oldest” remedy and thus the most known. 

In the treatment of depression, medication is only one issue; guidelines additionally focus on nonpharmacological treatments like psychotherapy, mind body techniques, or light therapy. These are also relevant therapeutic options which are very often underrepresented [23]. However, our data do not provide detailed information on such therapies. 

With regards to comorbidities, studies have shown the prevalence of depression to be higher for persons with heart diseases, diabetes mellitus, stroke, COPD, and cancer [28]. This was also confirmed in the study of Gill et al. 2008, which found depression to be more likely among patients with a significant number of medical comorbidities [21]. In our study, 504 (20.6%) had at least one comorbidity, while 204 patients (8.3%) had two or more comorbidities. This is nearly comparable to the proportions provided in the study of Gill et al. from 2010, who found 20.7% with one and 5.8% with two or more co-morbidities in their sample of 1513 patients [22]. They also found that after controlling for age and gender, patients with multiple comorbidities were less likely to be prescribed medication (adjusted odds ratio, 0.58; 95% CI, 0.35–0.96). In our first multivariate model, which equates the approach of Gill et al., we were also able to show this effect but were not able to reach significance (adjusted odds ratio 0.88; 95% CI, 0.71–1.09). A more detailed differentiation between the co-morbidities was not performed to guarantee the statistical model performance. 

Although there is some comparability of our results with former studies, some discrepancies of our results with another German study of Jacobi et al. [29] have to be mentioned. Although the proportion for one comorbidity with 20.8% is quite similar, they found 39.9% of depressive patients with two or more comorbidities. This may be explained by the fact that all patients with one depressive episode form the basis of their study which is not comparable with our situation.

## 5. Limitations

The present study has several important limitations which should be taken into account when interpreting the results. Firstly, additional data on the depression diagnoses are lacking. We do not know to what extent the diagnoses were made, only clinically or with additional validated questionnaires, that is, as a functional evaluation with the WHO-5 or PHQ-D [30, 31]. We therefore are also not able to give detailed information on the severity of the depression.

Secondly, although physician prescribing data were subjected to an internal review, coding inaccuracies cannot be ruled out entirely. 

Thirdly, our data do not provide more detailed information on the type and dosage of phytotherapeutic *Hypericum perforatum *preparations. For the same methodological boundaries, our data also do not allow a calculation of daily drug doses, which limits the comparability of our data with other studies.

Fourthly, data on subsequent medication use in patients who switched physicians were unavailable. 

Fifthly, our data from the group of 22 participating physicians are not representative for physicians in general practice in Germany nor may be seen as such for the smaller subgroup of anthroposophical physicians. The same problem arises for the patients the data are based on. Although an earlier paper gives an estimate for the prevalence of mood and affective disorders (F00-F39) of about 10% in our patients between 40 and 70 years in 2005 which is comparable to the numbers given, that is, in [7], it is less than the prevalence of 19.8% reported in [29]. Thus generalisations from this data are somehow limited. 

Finally, although there were no major differences to the studies of Gill et al., the present study lacks a direct comparison group and the options to carry out detailed subgroup analyses. Further research on this subject would benefit from including a comparison group of conventional primary care physicians.

## 6. Conclusion

This study provides a comprehensive analysis of everyday practice for treatment of depression in primary care in physicians with subspecialisation in anthroposophic medicine (AM). Although the administration of phytotherapeutic preparations from *Hypericum perforatum* was significantly higher, the prescribing frequency for conventional anti-depressive drugs is partly comparable to those found in other studies.

## Figures and Tables

**Table 1:** Characteristics of the study population according to co-morbidities, age, and gender.

Comorbid condition	Patients	Age group [years]			Gender [%]	
		<40	40–59	≥60	Male	Female
	*N* (%)	*n* (%)	*n* (%)	*n* (%)	*n* (%)	*n* (%)
Coronary heart disease	202	7 (3.5)	71 (35.1)	124 (61.4)	66 (32.7)	136 (67.3)
Cerebrovascular disease	99	—	21 (21.2)	78 (78.8)	26 (26.3)	73 (73.7)
Diabetes mellitus	174	9 (5.2)	54 (31.0)	111 (63.8)	66 (27.9)	108 (62.1)
Cancer	351	19 (5.4)	162 (46.2)	170 (48.4)	81 (23.1)	270 (76.9)
Congestive heart failure	113	1 (0.9)	12 (10.6)	100 (88.5)	23 (20.4)	90 (79.6)
Chronic obstructive pulmonary disease	68	8 (11.8)	25 (36.5)	35 (51.5)	19 (27.9)	49 (72.1)

Comorbidities						
0	1736	614 (35.4)	921 (53.1)	201 (11.6)	358 (20.6)	1378 (79.4)
1	504	40 (7.9)	257 (51.0)	207 (41.1)	142 (28.2)	362 (71.8)
≥2	204	2 (1.0)	40 (19.6)	162 (79.4)	55 (27.0)	149 (73.0)

Total	2444	656 (26.8)	1218 (49.8)	570 (23.3)	555 (22.7)	1889 (77.3)

**Table 2:** Top 10 of prescribed antidepressants.

Rank	Substance	ATC	Type	*N*	%	Cum %
1	*Hypericum perforatum *	N06AP	Phytoceutical^1^	1180	44.6	44.6
2	Amitriptyline	N06AA09	NSMRI	426	16.1	60.7
3	Mirtazapine	N06AX11	NaSSA	200	7.6	68.3
4	Citalopram	N06AB04	SSRI	197	7.4	75.7
5	Doxepin	N06AA12	NSMRI	140	5.3	81.0
6	Opipramol	N06AA05	TCAs^2^	104	3.9	85.0
7	Venlafaxine	N06AX16	SSNRI	69	2.6	87.6
8	Trimipramine	N06AA06	NSMRI	52	2.0	89.5
9	Fluoxetine	N06AB03	SSRI	51	1.9	91.5
10	Paroxetine	N06AB05	SSRI	46	1.7	93.2
11–28	Other < 1.7%^3^			138	9.4	100.0

Total				2645	100.0	100.0

MAOIs: nonselective, monoamine oxidase A inhibitors; NaSSAs: noradrenergic and specific serotonergic antidepressants; NSMRIs: non-selective monoamine reuptake inhibitors; TCAs: tricyclic antidepressants; SSRIs: selective serotonin reuptake inhibitors; SSNRIs: selective serotonin and noradrenergic reuptake inhibitors.

^1^Two of the primary active constituents of *Hypericum perforatum* are hyperforin and adhyperforin. Hyperforin and adhyperforin are wide-spectrum inhibitors of the reuptake of serotonin, noradrenaline, glutamate, dopamine, and GABA.

^2^Although opipramol is a member of the tricyclic antidepressants, today it is typically used in the treatment of generalized anxiety disorders (GAD).

^3^Others drugs: for example, Bupropion (NDRIs: noradrenergic and dopaminergic reuptake inhibitors) and nefazodone (DSAs: dual serotonergic antidepressants).

**Table 3:** Sample of patients with depression subdivided according to antidepressants.

	Patients	Antidepressant^1^					
	*N*	Any	NSMRI	SSRI	MAOI	Hyp.-perf.	Other^2^
		[*n* (%)]	[*n* (%)]	[*n* (%)]	[*n* (%)]	[*n* (%)]	[*n* (%)]
Gender							
Male	555	202 (36.4)	53 (9.5)	34 (6.1)	1 (0.2)	124 (22.3)	26 (4.7)
Female	1889	631 (33.4)	182 (9.6)	106 (5.6)	3 (0.2)	415 (22.0)	59 (3.1)
Age [years]							
<40	656	182 (27.7)	27 (4.1)	23 (3.5)	—	147 (22.4)	9 (1.4)
40–59	1218	398 (32.7)	109 (8.9)	73 (6.0)	2 (0.2)	260 (21.3)	41 (3.4)
≥60	570	253 (44.4)	99 (17.4)	44 (7.7)	2 (0.4)	132 (23.2)	35 (6.1)
Physician specialization							
GP	1793	522 (29.1)	124 (6.9)	83 (4.6)	2 (0.1)	355 (19.8)	44 (2.5)
Internist	437	148 (33.9)	27 (6.2)	26 (5.9)	1 (0.2)	110 (25.2)	6 (1.4)
Neurology	214	163 (76.2)	84 (39.3)	31 (14.5)	1 (0.5)	74 (34.6)	35 (16.4)
Type of depression							
Depressive episode	2158	762 (35.3)	219 (10.1)	120 (5.6)	4 (0.2)	497 (23.0)	78 (3.6)
Recurrent depressive disorder	286	71 (24.8)	16 (5.6)	20 (7.0)	—	42 (14.7)	7 (2.4)
Multi-comorbidity							
No	2240	737 (32.9)	202 (9.0)	122 (5.4)	3 (0.1)	492 (22.0)	69 (3.1)
Yes	204	96 (47.1)	33 (16.2)	18 (8.8)	1 (0.5)	47 (23.0)	16 (7.8)
Comorbidity							
Coronary heart disease	202	84 (41.6)	27 (13.4)	17 (8.4)	1 (0.5)	48 (23.8)	9 (4.5)
Cerebrovascular disease	99	56 (56.6)	27 (27.3)	13 (13.1)	—	23 (23.2)	12 (12.1)
Diabetes mellitus	174	82 (47.1)	23 (13.2)	15 (8.6)	1 (0.6)	46 (26.4)	10 (5.7)
Cancer	351	108 (30.8)	27 (7.7)	17 (4.8)	1 (0.3)	70 (19.9)	12 (3.4)
Congestive heart failure	113	59 (52.2)	22 (19.5)	11 (9.7)	1 (0.9)	30 (26.5)	6 (5.3)
Chronic obstructive pulmonary disease	68	34 (50.0)	11 (16.2)	3 (4.4)	1 (1.5)	19 (27.9)	8 (11.8)

Total	2444	833 (34.1)	235 (9.6)	140 (5.7)	4 (0.2)	539 (22.1)	85 (3.5)

^1^Double entries possible, ^2^including bupropion, mirtazapine, and nefazodone.

MAOIs: non-selective monoamine oxidase A inhibitors.

NSMRI: non-selective monoamine reuptake inhibitors.

SSRIs: selective serotonin reuptake inhibitors.

Others: for example, bupropion, mirtazapine, and nefazodone.

**Table 4:** Likelihood of being prescribed any antidepressant medication by co-morbidity (*n* = 2444).

Co-morbid condition	Patients who were prescribed an antidepressant		Likelihood of being prescribed antidepressant		
	Patients with co-morbidity	Patients without co-morbidity	Unadjusted OR	Model 1 Adjusted OR^1^ (95% CI)	Model 2 Adjusted OR^1^ (95% CI)
	*n*/*N* (%)	*n*/*N* (%)			
Coronary heart disease	84/202 (41.6)	749/2242 (33.4)	1.419 (1.058–1.903)*	1.028 (0.753–1.404)	1.191 (0.864–1.643)
Cerebrovascular disease	56/99 (56.6)	777/2345 (33.1)	2.628 (1.750–3.947)*	1.781 (1.159–2.739)*	1.762 (1.124–2.762)*
Diabetes mellitus	82/174 (47.1)	751/2270 (33.1)	1.803 (1.322–2.459)*	1.342 (0.968–1.860)	1.317 (0.936–1.855)
Cancer	108/351 (30.8)	725/2093 (34.6)	0.829 (0.657–1.070)	0.652 (0.505–0.842)*	0.745 (0.572–0.969)*
Congestive heart failure	59/113 (52.2)	774/2331 (33.2)	2.198 (1.504–3.211)*	1.431 (0.951–2.154)	1.652 (1.082–2.521)*
Chronic obstructive Pulmonary disease	34/68 (50.0)	799/2376 (33.6)	1.974 (1.218–3.199)*	1.612 (0.986–2.638)	1.950 (1.188–3.200)*
Any comorbidity	267/708 (37.7)	566/1736 (32.6)	1.252 (1.043–1.502)*	0.878 (0.709–1.086)	1.007 (0.807–1.257)

^1^Odds ratio for patients who had a co-morbidity compared to patients who did not have co-morbidity.

Model 1: adjusted for patient age and gender.

Model 2: adjusted for patient age and gender, as well as for physician specialisation and type of depression.
